# Prognostic Factors and Complications in Patients With Operational Peptic Ulcer Perforation in Northern Thailand

**DOI:** 10.14740/gr530w

**Published:** 2014-03-14

**Authors:** Chutikarn Suriya, Nongyao Kasatpibal, Wipada Kunaviktikul, Toranee Kayee

**Affiliations:** a Clinical Epidemiology Unit, Faculty of Medicine, Chiang Mai University, Chiang Mai, Thailand; b Faculty of Nursing, Chiang Mai University, Chiang Mai, Thailand; c Department of Surgery, Nakornping Hospital, Chiang Mai, Thailand

**Keywords:** Peptic ulcer perforation, Prognostic indicator, Post-operative complications

## Abstract

**Background:**

Peptic ulcer perforation (PUP) is a very serious condition that leads to excessive complications and mortality. This study aimed to explore the possible prognostic factors and complications in patients with perforated peptic ulcer operation.

**Methods:**

A 6-year retrospective cohort study in Nakornping Hospital between January 1, 2005 and December 31, 2010 was conducted. The study included 912 patients who underwent PUP surgery. Patient characteristics were analyzed by using frequency, percentage, mean (standard deviation) and median (range). A comparison between groups was made. The Pearson’s Chi-squared or Fisher’s exact test was used for categorical variables, as appropriate. The Student’s t test was used for continuous variables with normal distribution, and Wilcoxon rank sum test was performed for continuous variables with non-normal distributions. Exponential risk regression analysis was performed to estimate the relative risk (RR) for the prognostic factors with a probability value of < 0.05 as a statistically significant value. Post-operative length of stay was computed graphically based on Kaplan-Meier estimates.

**Results:**

During the study period, 912 post-operative PUP patients were observed. The median age of patients was 78.5 (15 - 92) years, and 77.74% of the patients were male gender. Multivariate analysis showed that five prognostic indicators: underlying illnesses; liver disease (RR: 5.41; 95% confidence interval (CI): 1.36 - 21.56) and kidney disease (RR: 4.72; 95% CI: 1.05 - 21.11); duration of operation > 3 h (RR: 9.83; 95% CI: 1.61-59.66); unplanned admission to ICU (RR: 9.22; 95% CI: 1.55 - 54.68); and prolonged ventilation > 24 h (RR: 9.02; 95% CI: 0.42 - 9.98) were associated with post-operative PUP complications. Post-operative complications developed in 87 (9.54%) patients with 135 complications: 11 (1.21%) patients underwent re-operation, 32 (3.51%) patients suffered with surgical site infection, 74 (8.11%) patients encountered with pneumonia and 18 (1.97%) patients died. Post-operative complications including surgical site infection (incidence rate ratio (IRR): 2.00; 95% CI: 0.76 - 5.27), re-operation (IRR: 2.65; 95% CI: 0.73 - 9.62) and pneumonia (IRR: 6.97; 95% CI: 6.30 - 7.70) tend to be associated with mortality. The risk ratio showed a trend towards an increased risk for post-operative mortality with smaller values. However, this trend was not statistically significant.

**Conclusions:**

The findings might have clinical importance as to optimize the surgical management of PUP and to minimize the complications or mortality.

## Introduction

The peptic ulcer perforation (PUP) is a serious problem and requires surgical treatment for repair of the perforation. Post-operative PUP complications are the leading cause of mortality worldwide [1-9]. Prognostic factors which influence post-operational complications are still matters of continuing debate. In the Western, the incidence of post-operative PUP complications varies with a wide range from 0.78 to 15% [4, 5, 9-13]. In Thailand, the incidence of post-operative PUP complications was limited. Limited publication presented overall complications in Siriraj Hospital, Mahidol University, Bangkok as mortality rate (9%) and the all complications rate (30%) [6]. Because they are life-threatening, complications [6, 10-13] of the PUP need appropriate treatment to prevent mortality. The purpose of this study was to determine the post-operative prognostic factors and complications in patients who have operation for PUP in Northern Thailand.

## Materials and Methods

A retrospective cohort study of consecutive 912 patients was conducted at a tertiary referral hospital, Nakornping Hospital in Chiang Mai. This has been served patients from connecting hospital around upper northern region Thailand between January 1, 2005 and December 31, 2010. The patients in whom histological diagnosis was confirmed as the malignant lesion in the stomach were excluded.

### Operation definition of variable

Post-operative PUP complications are conditions in which the patients have developed surgical site infection, need re-operation, have pneumonia or die in hospital, certified by surgeon, based on diagnostic criteria of the Center of Disease Control and Prevention [14].

### Data collection

Systematic chart review of patients with PUP was completed. This included demographic variables, history of underlying illness and clinical data. The prognostic factors and post-operative PUP complications were collected from admission to discharge.

### Statistical analysis

Patient characteristics were analyzed by using frequency, percentage, mean and standard deviation. A comparison between groups was made. The Pearson’s Chi-squared or Fisher’s exact test was used for categorical variables, as appropriate. The continuous variables were tested for normal distribution with the Kolmogorov-Smirnov test. The Student’s t test was used for continuous variables with normal distributions. Non-parametric test (Wilcoxon rank sum test) was used for continuous variables with non-normal distributions. Multivariable analysis using exponential risk regression analysis was performed to estimate the relative risk (RR) for the prognostic factors with a probability value of < 0.05 as a statistically significant value. Post-operative length of stay (PLOS) was computed graphically based on Kaplan-Meier estimates.

## Results

One thousand and twenty-six patients who underwent PUP operation were considered for enrollment. Among these subjects, 37 (3.61%) were ruled out with stomach cancer, 27 (2.63%) with inflammatory lesion or lymphoma lesions and 50 (4.87%) with uncompleted information. Nine hundred and twelve post-operative PUP patients were observed. The median age of patients was 72 (15 - 92) years. Seven hundred and nine patients (77.74%) were male gender. Eighty-seven patients (9.54%) had complications. Eight hundred and twenty-five patients (90.46%) recovered without complications. The median age of post-operative PUP patients with complication was 78.5 (15 - 92) years. One hundred and forty-two patients with underlying illnesses, 68 (78.16%) patients with systolic blood pressure ≤ 90 mmHg, 60 (68.97%) patients who referred from lower level hospitals, 33 (37.93%) patients who underwent duration of operation > 3 h with prolonged ventilation > 24 h and 70 (80.46%) patients with unplanned admission to ICU developed complications (Table 1).

**Table 1:** Patient Characteristics of Post-Operative PUP* Outcomes

Patient characteristics	Complications (n = 87, 9.54%)	Recovered (n = 825, 90.46%)	P value
Gender			
Male	64 (73.56)	645 (78.18)	0.325
Age (years)			
Median (IQR)	78.5 (15 - 92)	66 (15 - 87)	< 0.001
Underlying illnesses			
Diabetes mellitus	11 (12.64)	46 (5.58)	0.010
Hypertension	27 (31.03)	168 (20.36)	0.021
Lung disease	38 (43.68)	64 (7.76)	< 0.001
Liver disease	13 (14.94)	22 (2.67)	< 0.001
Heart disease	33 (37.93)	114 (13.82)	< 0.001
Kidney disease	20 (22.99)	57 (6.91)	< 0.001
Systolic blood pressure ≤ 90 mmHg	68 (78.16)	44 (5.33)	< 0.001
Referred from lower level hospitals	60 (68.97)	640 (77.58)	0.071
Duration of operation > 3 h	33 (37.93)	54 (6.55)	< 0.001
Prolonged ventilation > 24 h	33 (37.93)	54 (6.55)	< 0.001
Unplanned admission to ICU	70 (80.46)	630 (76.36)	0.390

* PUP: peptic ulcer perforation.

Post-operative complications developed in 87 (9.54%) patients with 135 complications: 11 (1.21%) patients underwent re-operation, 32 (3.51%) patients suffered with surgical site infection, 74 (8.11%) patients encountered with pneumonia and 18 (1.97%) patients died. Fifty-two patients had one complication, 25 patients had two, 7 patients had three and 3 patients had suffered from four complications (Table 2).

**Table 2:** Distribution of Post-Operational Complications

Post-operational complications	Number (n=912)	Percentage
Surgical site infection	32	3.51
Re-operation	11	1.21
Pneumonia	74	8.11
Death	18	1.97

Post-operational complications including surgical site infection (incidence rate ratio (IRR): 2.00; 95% confidence interval (CI): 0.76 - 5.27), re-operation (IRR: 2.65; 95% CI: 0.73 - 9.62) and pneumonia (IRR: 6.97; 95% CI: 6.30 - 7.70) tend to be associated with mortality. The risk ratio showed a trend towards an increased risk for post-operative mortality with smaller values. However, this trend was not statistically significant (Table 3).

**Table 3:** Frequency, Incidence Rate Ratio (IRR) and 95% Confidence Interval of Associated Outcomes With Mortality Between PUP Patients With and Without Post-Operational Complications

Associated outcomes	Outcomes presence	Mortality	IRR (95%CI)	P value
Surgical site infection	No	8 (44.44%)	Reference	0.158
	Yes	10 (55.56%)	2.00 (0.76 - 5.27)	
Re-operation	No	15 (82.33%)	Reference	0.137
	Yes	3 (16.67%)	2.65 (0.73 - 9.62)	
Pneumonia	No	0 (0.00%)	Reference	0.958
	Yes	18 (100.00%)	6.97 (6.30 - 7.70)	

The PLOS between patients with versus without post-operative PUP complications was a strong statistically significant linear relationship.

**Figure 1:**
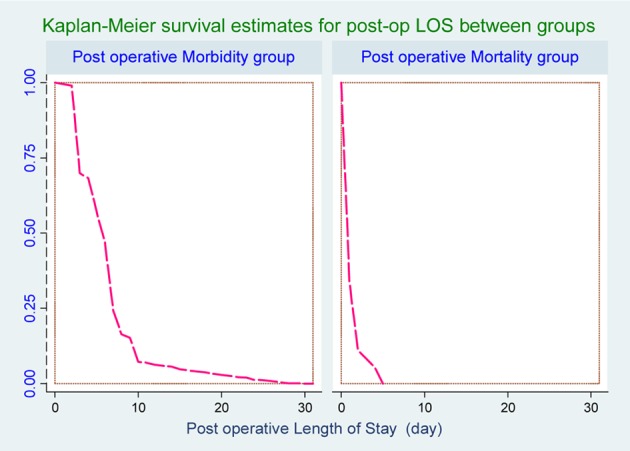
Survival curve for post-operative length of stay (PLOS) between groups.

The patients with another complication (surgical site infection, re-operation and pneumonia) had prolonged PLOS as much as 1-31 days. On the contrary, PLOS in hospital mortality patients was very short with 1-5 days (Table 4 and Fig. 1).

**Table 4:** Post-Operative Length of Stay (PLOS) Between PUP Patients With and Without Post-Operational Complications

Associated outcomes	PLOS (days)		P value
	Without complications (n = 825)	With complications (n = 87)	
Surgical site infection			
Median (IQR)	7 (1 - 29)	10 (7 - 31)	0.004
Re-operation			
Median (IQR)	6 (1 - 22)	11 (1 - 31)	0.002
Pneumonia			
Median (IQR)	6 (1 - 25)	15 (1 - 31)	< 0.001
Death			
Median (IQR)	6 (1 - 31)	1 (1 - 5)	< 0.001

Accordingly, multivariate analysis showed that five prognostic factors including underlying illnesses, liver disease (RR: 5.41; 95%CI: 1.36 - 21.56) and kidney disease (RR: 4.72; 95%CI: 1.05 - 21.11), duration of operation > 3 h (RR: 9.83; 95%CI: 1.61-59.66), unplanned admission to the ICU (RR: 9.22; 95%CI: 1.55 - 54.68) and prolonged ventilation > 24 h (RR: 9.02; 95%CI: 0.42 - 9.88) were associated with post-operative PUP complications (Table 5).

**Table 5:** Risk Ratio (RR) and 95% Confidence Interval of Prognostic Factors for Post- Operative Complications

Prognostic factors	Multivariable RR	95%CI	P value
Gender			
Male	0.35	0.06 - 1.96	0.235
Age (years)			
≥ 60	0.11	0.013 - 0.84	0.034
Underlining illnesses			
Diabetes mellitus	2.15	0.53 - 8.73	0.281
Diabetes mellitus	1.12	0.34 - 3.74	0.851
Lung disease	1.33	0.29 - 6.05	0.715
Liver disease	5.41	1.36 - 21.56	0.015
Heart disease	1.23	0.28 - 5.29	0.784
Kidney disease	4.72	1.05 - 21.11	0.042
Systolic blood pressure ≤ 90 mmHg	2.42	0.30 - 19.41	0.407
Duodenal ulcer perforation	3.52	0.31 - 40.50	0.313
Elective surgery	2.02	0.42 - 9.88	0.383
Duration of perforation > 3 h	9.83	1.61 - 59.66	0.013
Referred from lower level hospitals	0.97	0.18 - 5.10	0.969
Unplanned admission to ICU	9.22	1.55 - 54.68	0.014
Prolonged ventilation > 24 h	9.02	0.42 - 9.88	0.038

## Discussion

Patients with PUP had diffused from floods of the peritoneum with the acid contents of the stomach with more widespread spillage. This is a serious condition with acute inflammatory peritoneal reaction and trend to peritonitis when they were delayingly detected [15]. The decision-making of clinicians will be concerned with management outcome assisted by treatment guidelines. The surgical treatment is one of management in PUP patient.

In this study, five prognostic factors related to post-operative complications are underlying illnesses (liver and kidney disease), duration of operation > 3 h, unplanned admission to the ICU and prolonged ventilation > 24 h. These supported previous studies, factors associated with morbidity [4, 16-19] and some studies presented factors increased mortality after surgical treatment [1, 2].

The underlying illnesses (liver and kidney disease) in PUP patients are associated with morbidity because hepatic and renal functions are interconnected through both the existence of related primary organ and hemodynamic interrelationships. Renal and liver diseases caused renal and liver dysfunction. However, the presence and duration of renal and liver diseases before operation increase in association with prolonged operation time. They are risks of vascular and muscle necrosis with consequent renal failure and multiple organs failure. Multiple organs failure was identification of underlying pathogenic mechanisms and possible mediators of specific organ system failures, so that unplanned admission to the ICU therapy may be directed at the initial post-operative complications [15, 20].

The post-operative complications in PUP patients in this study included re-operation, surgical site infection, pneumonia and death during hospitalization. The result supports previous studies that morbidity and mortality rates of any complication of ulcer disease are around 10 to 15% [15, 20].

PLOS in patients with hospital mortality was shorter than morbidity group, within 1-5 days. They were admitted with severity conditions. It can be considered a main indicator of patients’ post-operative complications as a clinical indicator from the operating care and has been thought to reflect the quality of care.

### Study strengths

The strengths of the study were able to include a large number of patients. Furthermore, we were able to include all patients admitted with PUP who underwent open surgery.

### Study weakness

A limitation on this study was retrospectively reviewed database of medical information. As the data were reported previously, so they might be not in high novelty; in addition, the analysis of occurrence of post-operative complications was based on only surgeon’s decision, so multi-disciplinary would be granted.

### Conclusion

The protocols to reduce complications in PUP patients might be developed, which could classify patients into critical and non-critical groups. However, patients in critical group should be required urgent clinically suitable.
